# Primary pancreatic glomus tumor invading into the superior mesenteric vein: a case report

**DOI:** 10.1186/s40792-020-01058-7

**Published:** 2020-11-03

**Authors:** Ichiro Tamaki, Yohei Hosoda, Hironobu Sasano, Yu Sasaki, Hidenori Kiyochi, Yoshiro Taki, Izumi Komoto

**Affiliations:** 1 Department of Surgery, Kansai Electric Power Hospital, Fukushima 2-1-7, Fukushima-ku, Osaka City, Osaka 553-0003 Japan; 2 Graduate School of Medicine, Anatomic Pathology, Tohoku University, Sendai, Japan

**Keywords:** Glomus tumor, Glomangiomyoma, Pancreas, Immunohistochemistry, Surgical resection

## Abstract

**Background:**

Glomus tumors are subcutaneous tumors arising from glomus bodies, thermoregulatory components of the skin. These tumors could occur in visceral organs where glomus bodies are not normally present. Herein, we report a case of primary pancreatic glomus tumor with aggressive direct invasion into the superior mesenteric vein (SMV). To the best of our knowledge, this is the second case report of a glomus tumor arising in the pancreas.

**Case presentation:**

A 46-year-old woman was referred to our hospital due to vomiting with epigastric and back pain. Dynamic-CT revealed a well-circumscribed hypervascular mass, measuring 37 mm in its maximal diameter involving the pancreatic head. Both CT and endoscopic ultrasonography (EUS) revealed direct invasion into the SMV and radiologically suspected tumor thrombus. Biopsy sample obtained by EUS-guided fine needle aspiration revealed proliferation of small cells, round-to-oval tumor cells with round nuclei and scant cytoplasm. A histological diagnosis of pancreatic neuroendocrine tumor, G1 was initially considered. Therefore, subtotal stomach-preserving pancreatoduodenectomy using Child-II reconstruction was subsequently performed. Her SMV was resected and reconstructed due to extensive tumor involvement. Subsequent histopathological analysis revealed solid tumor cells proliferation that comprised oval-shaped nuclei and scant cytoplasm around disorganized or slit-shaped vessels in hematoxylin–eosin-stained slides. Immunohistochemical analysis then demonstrated positive immunoreactivity for smooth muscle actin, vimentin, and CD34, but negative for chromogranin A, synaptophysin, CD56, and signal transducer and activator of transcription 6. Based on these histological findings of resected specimens, the lesion was subsequently diagnosed as a primary pancreatic glomus tumor harboring direct invasion into the SMV. Her postoperative course was uneventful and annual surveys for the following 4 years post-op detected no clinical signs of recurrence.

**Conclusions:**

We report a very rare case of glomus tumor of the pancreas accompanied by venous invasion. Curative surgical resection is the best treatment option for pancreatic glomus tumors. Although pancreatic glomus tumor is rare, it should be taken into consideration in the differential diagnosis of a pancreatic solid tumor with hypervascularity.

## Background

Glomus tumors are rare mesenchymal neoplasms arising from the glomus body, a specialized form of arteriovenous anastomosis, mostly localized in dermal soft tissue and serves a thermoregulatory function [1]. Though extremely rare, glomus tumors in the visceral space could occur in any organ. The great majority of such cases generally possess benign histological features. However, cases of invasive glomus tumor have been also previously reported. Herein, we report a case of primary pancreatic glomus tumor that exhibited aggressive direct invasion of the superior mesenteric vein (SMV).

## Case presentation

### History of illness

A 46-year-old woman was admitted to a nearby clinic due to vomiting. She also had symptoms of epigastric and back pain noted after meals for 1-year duration. The patient further reported a 3-kg weight loss within 3 months prior to admission. Abdominal ultrasonography (US) revealed a solid tumor (4 cm in diameter) in the pancreatic head. She was then referred to our hospital for further management.

The patient had no past medical history or abdominal surgery. Her past medical history showed a previous abdominal US as part of a scheduled health check 5 years prior to admission, which revealed no abnormal findings at that time. Her family history revealed gastric cancer in her father.

### Clinical and cytological investigation

**Fig. 1:**
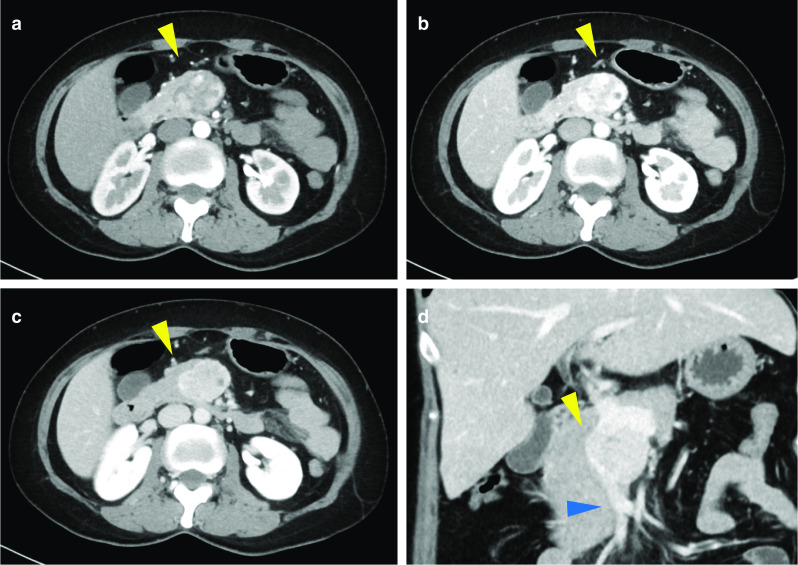
Dynamic-CT scan (**a** early arterial phase, **b** late arterial phase, **c** and **d** portal phase). Dynamic-CT scan revealing a hypovascularized tumor (yellow arrowhead) invading the superior mesenteric vein (blue arrowhead)

**Fig. 2:**
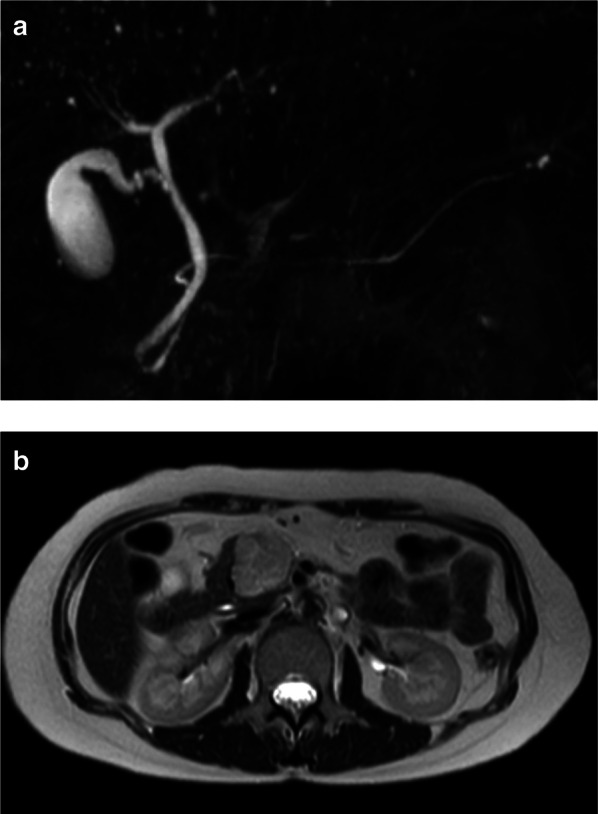
MR images. **a** Magnetic resonance cholangiopancreatography (MRCP) demonstrating an intact main pancreatic duct. **b** T2-weighted MR highlighting the tumor as a high signal area

**Fig. 3:**
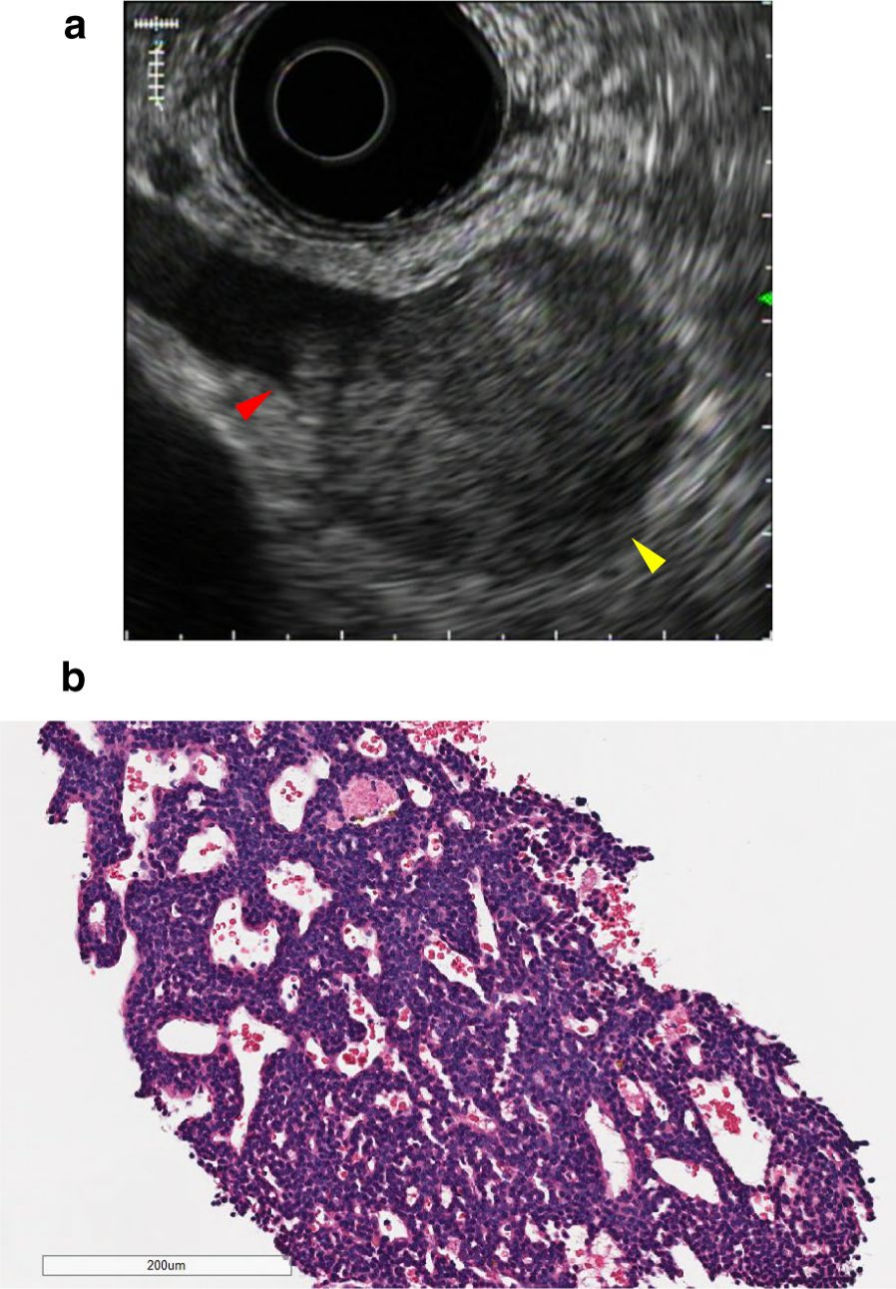
Endoscopic ultrasonography (EUS). **a** EUS illustrating a well-circumscribed round tumor (yellow arrowhead). An intravenous high echoic mass invading the SMV is shown adjacent to the main tumor (red arrowhead). **b** Hematoxylin–eosin (HE) staining of the EUS-FNA-obtained specimen exhibiting small, round-to-oval tumor cells with round nuclei and scant cytoplasm

Dynamic-CT study revealed a solid tumor, measuring 37 mm in its greatest diameter involving the pancreas head, showing dense enhancement in the arterial dominant phase that continued to the portal phase. The tumor vasculature was heterogeneous, and partial strong enhancement suggested the existence of developed vessels inside the tumor. An area of SMV encasement, measuring approximately 3 cm in axial length, indicated a massive direct tumor invasion of the SMV and possible tumor thrombus therein (Fig. 1). Magnetic resonance cholangiopancreatography (MRCP) demonstrated an intact main pancreatic duct (MPD). The tumor showed a high-signal-intensity area on T2-weighted MR images. MR images also exhibit well-circumscribed solid tumor invading the SMV (Fig. 2). Radiological studies found no signs of distant metastasis. Complete blood count (CBC) parameters were within the reference range. Blood serological analysis of carcinoembryonic antigen (CEA) and colorectal carcinoma antigen 19-9 (CA19-9) were also within the reference range. Serum aminotransferases, alkaline phosphatase, and total bilirubin levels were likewise, all normal. Endoscopic ultrasonography (EUS) revealed a well-circumscribed round tumor in the head of the pancreas. EUS also detected an intravenous high echoic mass in the SMV, suggesting the possibility of tumor thrombus or trans-luminal tumor penetration into the SMV wall (Fig. 3a). EUS-guided fine needle aspiration (EUS-FNA) yielded satisfactory biopsy sample containing small cells, round-to-oval tumor cells with round nuclei and scant cytoplasm (Fig. 3b). Ki-67 labeling index of the sample was 1.9%. Histological diagnosis was pancreatic neuroendocrine tumor (pan NET), G1 at that time. However, findings contradictory to the diagnosis of pan NET were also noted. Immunohistochemistry (IHC) of the biopsy sample revealed equivocal reactivity for synaptophysin and negative reactivity for chromogranin A expression. In addition, somatostatin receptor scintigraphy (Octreoscan®, Mallinckrodt Medical, Tokyo, Japan) revealed no radiotracer accumulation in the tumor.

### Treatment

**Fig. 4:**
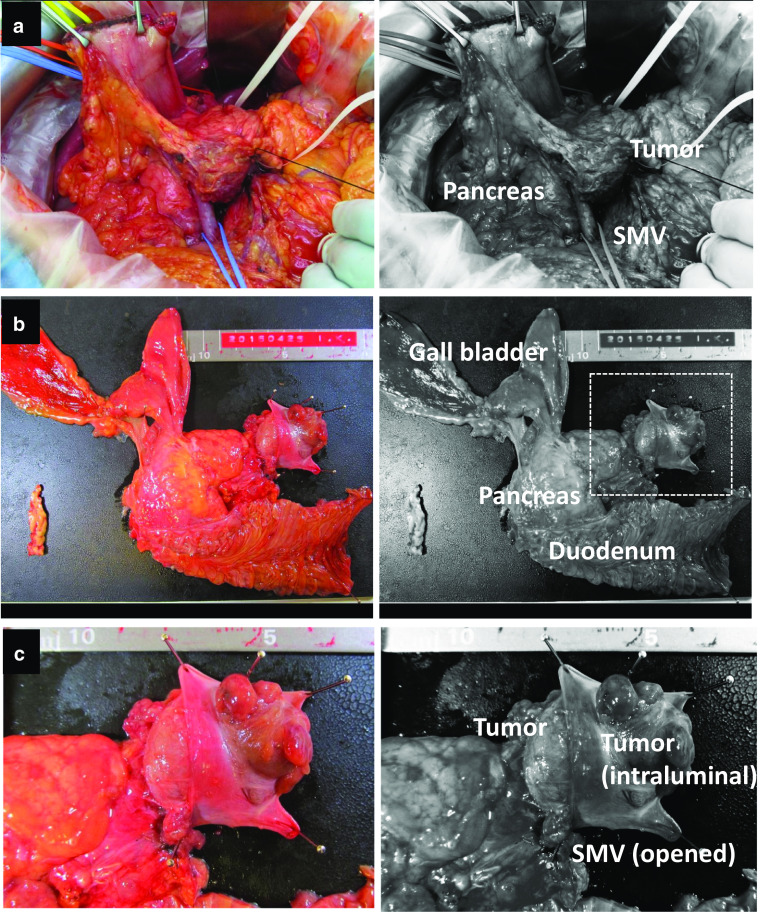
Gross specimen photographs. **a** Intraoperative photograph shows the main tumor, the superior mesenteric vein (SMV) and pancreas head. **b**, **c** Gross examination of the resected specimen showing a pancreatic tumor invading the SMV

Subtotal stomach-preserving pancreatoduodenectomy using Child-II reconstruction was performed. Intraoperative inspection revealed no signs of peritoneal dissemination or lymph node metastasis. Further intraoperative US examination demonstrated a well-circumscribed pancreatic tumor involving the SMV. The involved SMV was resected and reconstructed during surgery. Gross examination of the resected specimen revealed a well-circumscribed round soft tumor in the pancreatic head, 40 mm in diameter, penetrating the SMV wall with intraluminal tumor progression (37 mm in maximal length) (Fig. 4). The surgical margins were negative and the harvested lymph nodes were likewise negative for metastases both macroscopically and histologically.

The postoperative course was uneventful and the patient was discharged from hospital 15 days after surgery.

### Pathological findings

**Fig. 5:**
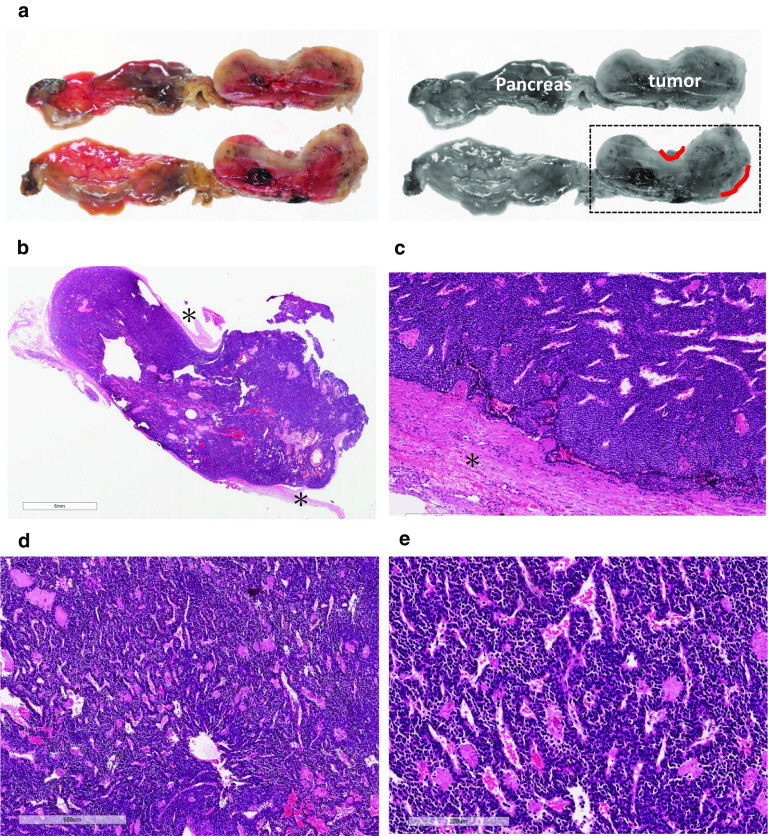
Histopathology. **a** Macroscopic findings of the tumor division surface after formalin fixation. The solid tumor arising from pancreas body invading the SMV wall (traced in red). An interrupted square shows the part shown in “b”. **b**, **c**, **d**, **e** Hematoxylin–eosin (HE) staining of the resected specimen revealed solid proliferation of tumor cells with oval-shaped nuclei and scanty cytoplasm around the disorganized or slit-shaped vessels. Asterisks indicate the SMV wall in the specimen

**Fig. 6:**
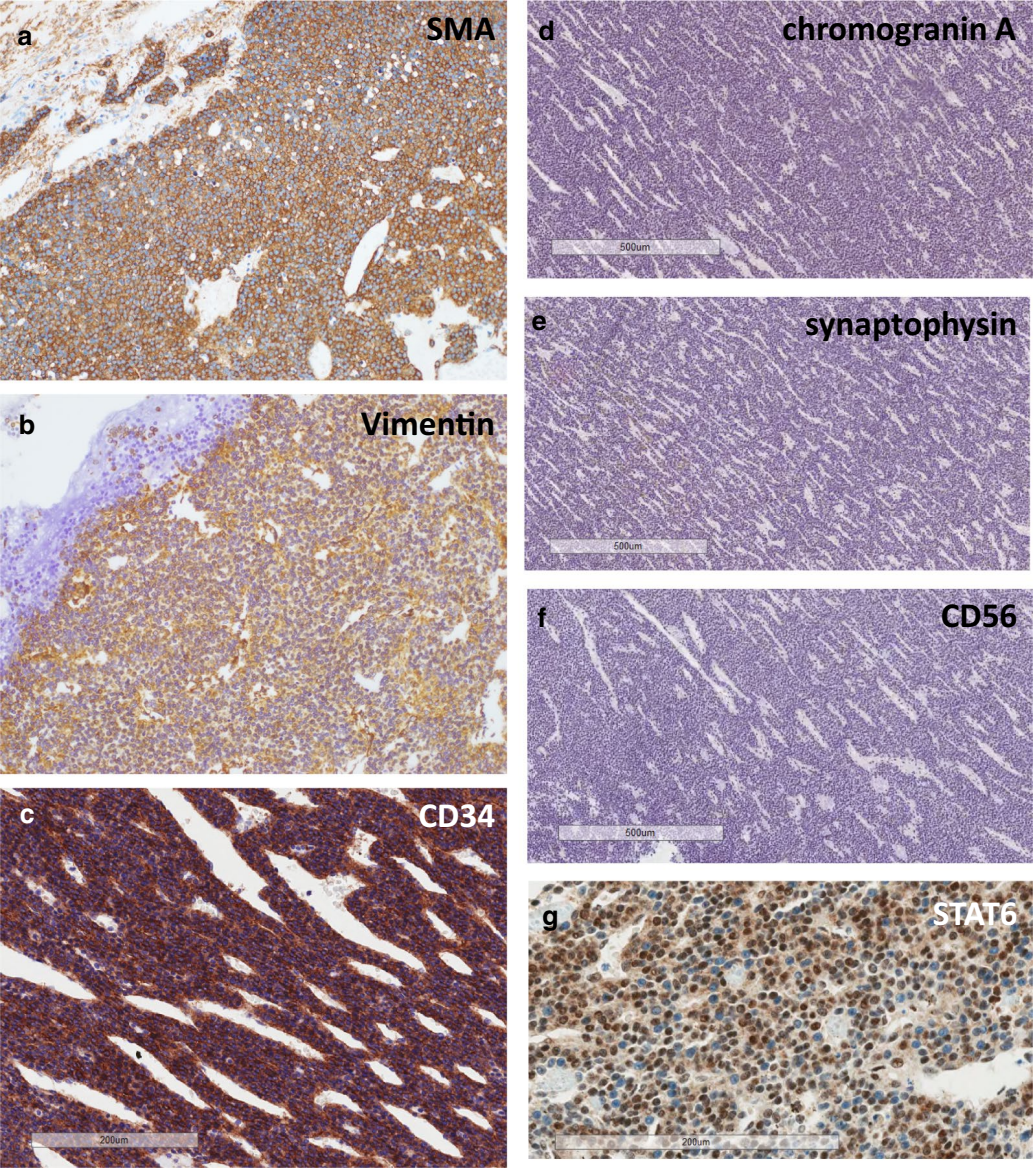
Immunohistochemistry. Immunohistochemistry demonstrates positive reactivity for smooth muscle actin (**a**), vimentin (**b**), and CD34 (**c**). Chromogranin A (**d**), synaptophysin (**e**), CD56 (**f**), and STAT6 (**g**) show negative reactivity, respectively

Tumor division surface represented the solid tumor penetrating the SMV wall (Fig. 5a). Hematoxylin–eosin (HE) staining of the resected specimen revealed solid proliferation of tumor cells with oval-shaped nuclei and scanty cytoplasm around disorganized or slit-shaped vessels along with glomangiopericytomatous differentiation. Tubular–lobular architecture was not histologically apparent in the specimen examined, indicating that the tumor was not of pancreatic ductal/acinar cell origin (Fig. 5b–e). IHC demonstrated positive immunoreactivity for smooth muscle actin (Fig. 6a), vimentin (Fig. 6b), and CD34 (Fig. 6c). IHC also showed negative immunoreactivity for chromogranin A, synaptophysin, and CD56 (Fig. 6d–f, respectively). These findings eliminated pan NETs from differential diagnosis. IHC of signal transducer and activator of transcription 6 (STAT6), which has been used to differentiate solitary fibrous tumors from other soft tissue tumors [2], yielded negative immunoreactivity (Fig. 6g). Therefore, this lesion was finally diagnosed as a primary pancreatic glomus tumor. The Ki-67 labeling index was 2% at hot spots.

Throughout the follow-up period, the patient was in good health with an annual CT study detecting no signs of recurrence for 4 years post-operatively.

## Discussion

Glomus bodies are thermoregulatory arteriovenous shunts in the deeper dermis mostly concentrated in peripheral sites such as the skin of fingertips. Glomus tumors arise from smooth muscle cells of the glomus bodies [3]. A glomus tumor typically occurs in subcutaneous tissues and presents with a triad of localized tenderness, marked paroxysmal pain, and sensitivity to cold [4]. However, glomus tumors could also occur in regions where glomus bodies do not normally exist [1]. A small number of case reports have illustrated visceral glomus tumors involving the stomach [5–7], small intestine [8, 9] and lung [10]. Miliauskas et al*.* reported the first case of pancreatic glomus tumor in a 17-year-old girl in 2002 [11]. A PubMed search using the keywords of “Glomus tumor” and “pancreas” confirmed that our present case represents the second reported case of primary pancreatic glomus tumor in the literature.

As previously mentioned, the preoperative diagnosis was pan NET G1, based on dynamic-CT images. These images showed a well-circumscribed tumor with marked enhancement and cytological findings of proliferation of small cells, round-to-oval tumor cells obtained by EUS-FNA. However, IHC findings were inconsistent with pan NET, which made us consider other possible entities prior to surgery. Previous reports have suggested that glomus tumors in abdominal cavity are sometimes misdiagnosed as gastrointestinal stromal tumors (GISTs) [5, 8] or NET [7] preoperatively. In this present case, histological features of the surgically resected specimen indicated the specific appearance of glomus tumor as previously described. In addition, the IHC profiles were also consistent with those of previously reported glomus tumors. A retrospective analysis of the previously obtained EUS-FNA findings further confirmed our final diagnosis of pancreatic glomus tumor as it exhibited histological characteristics specific to that tumor type.

Regarding biological behavior, the great majority of glomus tumors are benign. However, approximately 1% of glomus tumors are reported to be malignant [1, 10]. In our present patient, the tumor had aggressive invasion into the SMV, penetrating the venous wall and even exhibiting intraluminal tumor growth. Folpe et al. examined 52 unusual glomus tumors previously diagnosed as “atypical” or “malignant” in terms of nuclear atypia, infiltrative growth, or mitotic activity. In their study, they defined “malignant” glomus tumor as: tumors with a deep location and a size of more than 2 cm, or atypical mitotic figures, moderate-to-high nuclear grade, or ≥ 5 mitotic figures/50 HPF. There is a marked risk of metastasis and mortality in patients whose tumors meet their criteria [12]. In our present case, the tumor was located in the pancreas and measured 40 mm in its greatest diameter. In addition, the tumor directly invaded into the SMV. Therefore, no atypical mitotic figures were histologically identified. However, this case was still considered to harbor malignant potential according to Folpe’s criteria. Due to this malignant potential, the patient underwent close observation for 4 years following a curative resection.

Lastly, concerning the radiological characteristics of visceral glomus tumors, dynamic-CT exhibits dense enhancement on the arterial phase and continuous enhancement on the delayed phase. However, these findings are not specific to glomus tumors. GIST and neuroendocrine neoplasms show similar patterns [6].

In conclusion, we report a very rare case of glomus tumor in the pancreas exhibiting massive venous invasion. Curative surgical resection remains the best treatment option for pancreatic glomus tumors. Although it is a rare neoplasm, it should be taken into consideration when making a differential diagnosis of a pancreatic solid tumor with hypervascularity.

## Data Availability

Not applicable.

## Abbreviations

CA19-9: Colorectal carcinoma antigen 19-9
CBC: Complete blood count
CEA: Carcinoembryonic antigen
EUS: Endoscopic ultrasonography
EUS-FNA: EUS-guided fine needle aspiration
HE: Hematoxylin–eosin
IHC: Immunohistochemistry
MPD: Main pancreatic duct
MRCP: Magnetic resonance cholangiopancreatography
pan NET: Pancreatic neuroendocrine tumor
SMV: Superior mesenteric vein
US: Ultrasonography
